# The elusive evidence for chromothripsis

**DOI:** 10.1093/nar/gku525

**Published:** 2014-06-17

**Authors:** Marcus Kinsella, Anand Patel, Vineet Bafna

**Affiliations:** 1 Bioinformatics and Systems Biology Program, University of California, San Diego, CA, USA; 2 Department of Computer Science and Engineering, University of California, San Diego, CA, USA

## Abstract

The chromothripsis hypothesis suggests an extraordinary one-step catastrophic genomic event allowing a chromosome to ‘shatter into many pieces’ and reassemble into a functioning chromosome. Recent efforts have aimed to detect chromothripsis by looking for a genomic signature, characterized by a large number of breakpoints (50–250), but a limited number of oscillating copy number states (2–3) confined to a few chromosomes. The chromothripsis phenomenon has become widely reported in different cancers, but using inconsistent and sometimes relaxed criteria for determining rearrangements occur simultaneously rather than progressively. We revisit the original simulation approach and show that the signature is not clearly exceptional, and can be explained using only progressive rearrangements. For example, 3.9% of progressively simulated chromosomes with 50–55 breakpoints were dominated by two or three copy number states. In addition, by adjusting the parameters of the simulation, the proposed footprint appears more frequently. Lastly, we provide an algorithm to find a sequence of progressive rearrangements that explains all observed breakpoints from a proposed chromothripsis chromosome. Thus, the proposed signature cannot be considered a sufficient proof for this extraordinary hypothesis. Great caution should be exercised when labeling complex rearrangements as chromothripsis from genome hybridization and sequencing experiments.

## INTRODUCTION

In a groundbreaking 2011 study (1), Stephens *et al.* observed a pattern of structural variation in a leukemia genome so atypical it presumptively revealed a novel mechanism of chromosome rearrangement. Two features distinguish this variation pattern. First, the chromosome or chromosomal region in question has many clustered breakpoints that suggest complex adjacencies rather than simple deletions or non-overlapping tandem duplications. Second, the region oscillates between two or perhaps three copy number states.

To further investigate this phenomenon, Stephens *et al.* sequenced several cell lines with chromosomes that exhibited these features. One of these chromosomes was chromosome 15 from SNU-C1, a colon cancer cell line. This chromosome has 239 breakpoints identified by paired-end sequencing (PES) and mostly oscillates between two copy number states, two and four. Using simulations, Stephens *et al.* showed that the progressive introduction of the breakpoints they observed would result in a chromosome with many copy number states rather than just two. They hypothesized that the peculiar rearrangement pattern was not the result of progressive rearrangements but instead the result of the chromosome shattering followed by the random stitching together of the resulting pieces. They termed this phenomenon ‘chromothripsis’.

To determine how widespread chromothripsis may be, Stephens *et al.* used the progressive rearrangement simulation from SNU-C1 to conclude that a chromosome with at least 50 breakpoints dominated by at most three copy number states was unlikely to have been rearranged progressively and thus was likely to be a product of chromothripsis. Using these criteria they searched copy number profiles and estimate 2–3% of cancers have a chromosome that bears the hallmark of chromothripsis.

This is a striking result; it suggests a mechanism of cancer genome evolution that contrasts starkly with previously described models. This discovery has generated excitement and ongoing investigation. Subsequent studies have found evidence for chromothripsis in multiple myeloma (2), medulloblastoma (3,4), neuroblastoma (5) and colorectal cancers (6) as well as the germline (7,8). Moreover in some studies, chromothripsis has been associated with more aggressive cancers. Thus, it would appear that a new source of human disease has been found, with potentially far-reaching effects on our understanding and treatment of cancer (9).

The great potential of chromothripsis cannot be realized unless it can be accurately detected. It is unlikely that chromothripsis will ever be reliably observed directly, so we will need to rely on the footprint that chromothripsis should leave in copy number and breakpoint data. The characterization of this footprint is an open problem. While Stephens *et al.* searched for chromosomes dominated by at most three copy number states with at least 50 positions where copy number changes, subsequent works have used more relaxed criteria. They have required fewer breakpoints per chromosome, such as 20 (5), 10 (3,4) or just a handful (8). They also have not always required that the number of unique copy states in a chromosome be limited to two or three (4,5,10).

The validity of these footprints of chromothripsis rests on the idea that progressive rearrangement cannot create such patterns. However, the evidence for this proposition is largely limited to the initial simulation work by Stephens. Chromothripsis is now being investigated in different contexts than Stephens’ cell line simulations. Furthermore, the diversity of approaches used to identify chromothripsis means some groups are likely over- or underestimating its prevalence. This, together with the potentially great significance of chromothripsis, highlights the value of revisiting and extending the simulation work that underlies current strategies for identifying chromothripsis.

In this article, we review the simulation approach that suggests that progressive rearrangements cannot yield a chromosome with many breakpoints and few unique copy number states. First, we explore whether changes to the implementation of the simulation affect the validity of the footprint of chromothripsis. We show that a subtle but consequential error in the original implementation of the simulation causes it to understate the breakpoint and copy number patterns that can be achieved by progressive rearrangement. We examine varying possible meanings of ‘breakpoint’ and ‘copy number state’ and determine definitions that more closely correspond to experimental results. Next, we show that progressive rearrangement with a preference for inversions can produce chromosomes that bear the putative footprint of chromothripsis. Together these issues suggest that, assuming the simulation approach is valid, more stringent criteria must be used to identify chromothripsis and that the current literature overstates its prevalence.

We then demonstrate that the simulation approach produces similar results whether a chromosome is progressively rearranged or not. This undermines its ability to distinguish between chromothripsis and progressive rearrangement. Extending on this finding, we demonstrate a method that finds plausible progressive rearrangements that explain the breakpoints of particular chromosomes that have been documented to have undergone chromothripsis. Finally, we discuss the significance of these findings and question the chromothripsis hypothesis.

## MATERIALS AND METHODS

### Simulating progressive rearrangements

**Figure 1.**
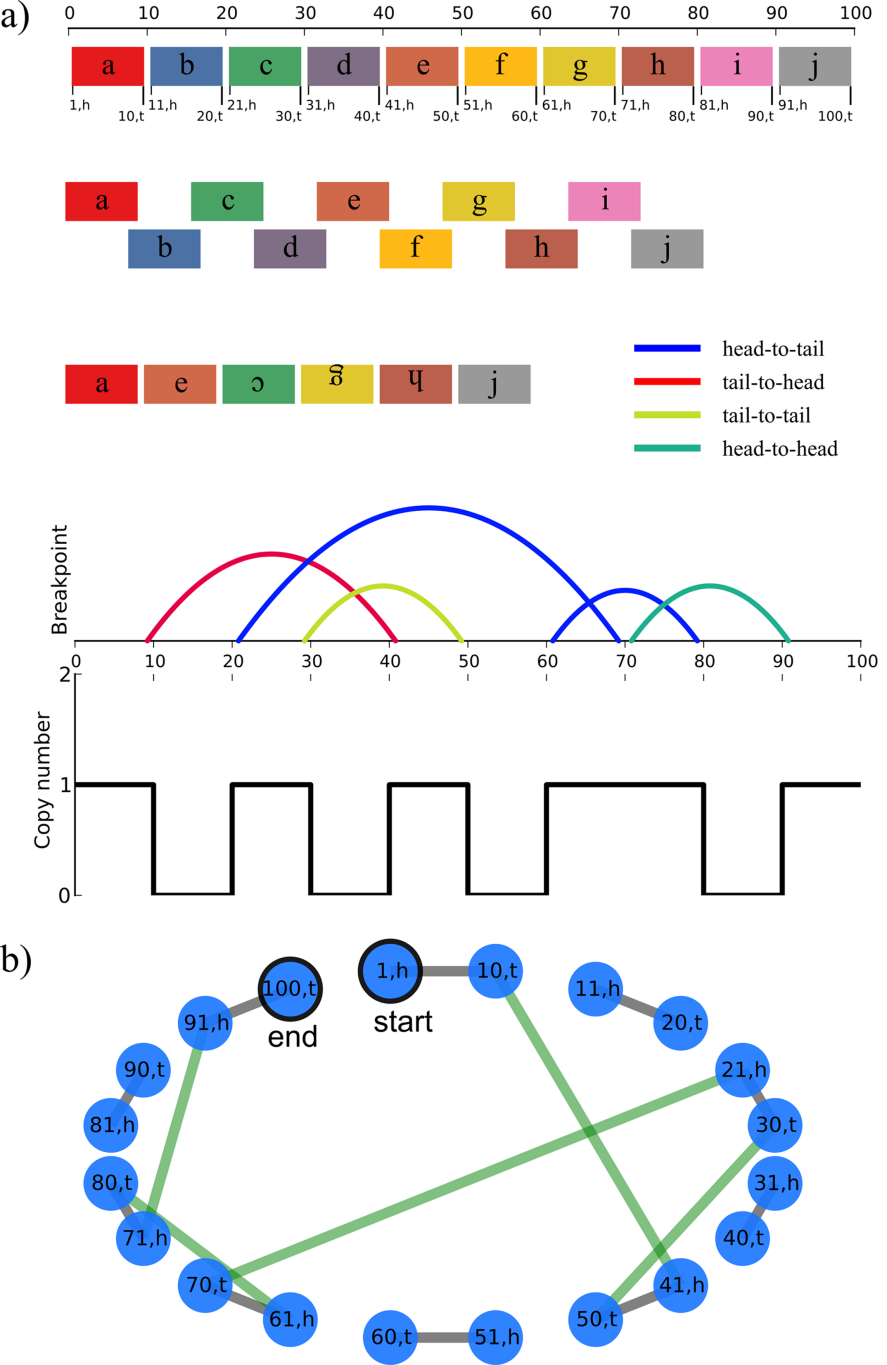
A hypothetical chromosome of length 100 shattered into blocks of length 10 and then reassembled. The hypothetical rearranged chromosome breakpoints are in Table 1. (**a**) The breakpoints and block copy number projected onto original chromosome. (**b**) A breakpoint graph representing the hypothetical rearranged chromosome with Table 1 breakpoints.

We will first summarize the simulation method. Consider a chromosome 100 bases long that undergoes chromothripsis, shattering into 10 segments of 10 bases, which we label a through j. The segments come back together, but some are lost, some are inverted and the order is shuffled. Suppose the resulting arrangement of segments is ae(-c)(-g)(-h)j. If this chromosome is sequenced, it will reveal five breakpoints and copy numbers that alternate between zero and one. The breakpoints are shown in Table 1, and an illustration of breakpoint positions is shown in Figure 1a.

**Table 1.** Breakpoints of a rearranged chromosome in Figure 1

Lower position	Lower segment	Terminus of lower segment	Higher position	Higher segment	Terminus of higher segment
10	a	tail	40	e	head
30	c	tail	50	e	tail
20	c	head	70	g	tail
60	g	head	80	h	tail
70	h	head	90	j	head

**Figure 2.**
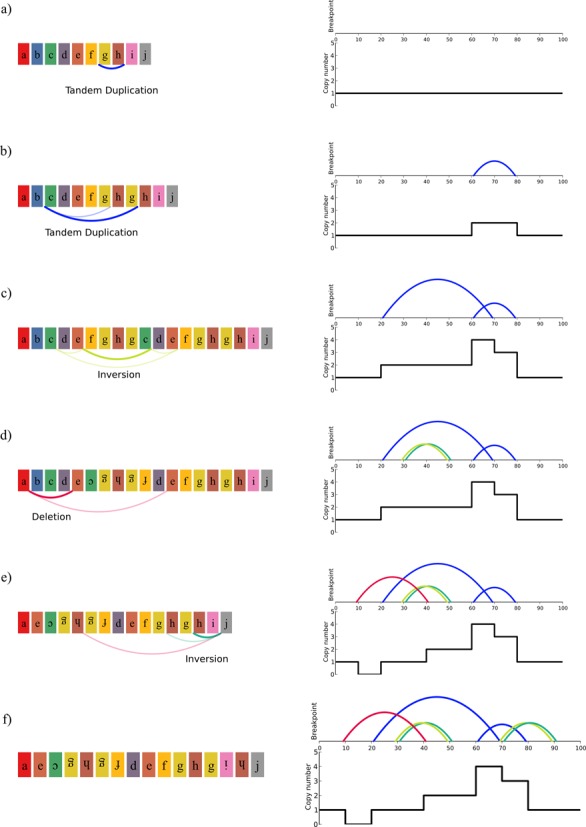
Following Stephens *et al.* simulation procedure, a sequence of possible rearrangements steps to explain the observed breakpoints in Table 1.

We can now step through the progressive rearrangement simulation used by Stephens *et al.* The simulated chromosome begins intact, with no rearrangements (Figure 2a). Then, a random breakpoint is chosen from the set of observed breakpoints. In this case, suppose the breakpoint between 60 and 80 is chosen. This breakpoint is now introduced into the chromosome via one of three rearrangement types: inversion, deletion or tandem duplication. The observed orientation of the two ends of the breakpoint is head-to-tail. So, an inversion cannot be used to create the breakpoint because that will result in segments with orientations of head-to-head or tail-to-tail. A deletion between 60 and 80 will not work because then the orientations would be tail-to-head. But, a tandem duplication between 60 and 80 will result in a breakpoint that, when read from 60 to 80, will join the head of segment g to the tail of segment h. So, segments g and h are duplicated. Suppose, the breakpoint between 20 and 70 is chosen and segments c to the furthest segment g are duplicated. Next, the rearrangement between 30 and 50 is chosen. Using similar reasoning as above, a rearrangement that reproduces the breakpoint is introduced. Suppose the rearrangement chosen is an inversion of segments fghgc. Note that this creates two breakpoints, the observed breakpoint plus another one in head-to-head orientation. Then, two more rearrangements are introduced resulting in the chromosome in Figure 2f. The number of breakpoints and copy number states in this chromosome would be recorded, and the simulation would be repeated many times with different rearrangement orders and segment choices. It would also be stopped when specific numbers of breakpoints had been introduced so that the relationship between the number of breakpoints and the number of unique copy number states could be determined.

### Finding chromosome rearrangements consistent with observed breakpoints

In addition to simulations, we can propose sequences of rearrangements that explain all the observed breakpoints. Each breakpoint is provided as a pair of coordinates that is non-adjacent in the reference genome, but adjacent in the donor sample. We first construct a *breakpoint graph* (11) from breakpoints (e.g. Figure 1b graph is constructed from breakpoints in Table 1): Partition the chromosome of length *L* into *n* segments based on breakpoint coordinates *p*_1_, *p*_2_, …. Each segment has two ends labeled by their position on the chromosome with the 5′ segment marked as *head (h)* and the 3′ segment end marked as *tail (t)*. The breakpoint graph is described by 2*n* nodes, denoted {(*p*_0_, *h*), (*p*_1_, *t*), (*p*_1_, *h*), (*p*_2_, *t*), …, (*p*_2*n* − 1_, *t*), (*p*_2*n* − 1_, *h*), (*p*_2*n*_, *t*)}, where *p*_0_ = 1 and *p*_2*n*_ = *L*. Pairs of nodes are connected by *segment-edges* and *breakpoint-edges*. Segment-edges (shown in grey in Figure 1b) connect (*p*_*i* − 1_, *h*) to (*p*_*i*_, *t*) for all 1 ≤ *i* ≤ 2*n*. Thus, every node has exactly one segment edge. Recall that the input is a set of breakpoints, 〈(*p*_*i*_, *e*_*i*_), (*p*_*j*_, *e*_*j*_)〉, …, where *p*_*i*_, *p*_*j*_ correspond to the chromosomal positions that are brought together, and *e*_*i*_, *e*_*j*_ are each either *head* or *tail*. By definition, the breakpoint graph already has the nodes (*p*_*i*_, *e*_*i*_) and (*p*_*j*_, *e*_*j*_), and we connect each such pair with a *breakpoint-edge* (shown in green). When constructing the graph from the Stephens *et al.* data, no node had more than one breakpoint edge. The observed loss of heterozygosity in chromosomes suggested that a single chromosome undergoes rearrangement. Our method uses the haploid assumption, by finding a single path in the breakpoint graph. Alternatively, finding two disjoint paths in the breakpoint graph corresponds to two rearranged chromosomes, without changing the observed breakpoints.

Based on sorting of reversals theory (11), a continuous path from the start node to the end node reads out a sequence of alternating segment and breakpoint edges representing the rearranged chromosome. Given the continuous path, there is always a sequence of reversals (inversions) that transforms the rearranged chromosome back into the original segment ordering. In Figure 1b, the only graph component of this type is the path from (1,h) to (100,t). If the set of observed breakpoints is not complete, we will have multiple connected components. This is remedied by chaining paths together with new breakpoint edges. The termini of paths with breakpoint edges are nodes with a single outgoing segment edge. To create a full path between chromosome start and end nodes, begin with a path containing the start node and connect the non-start terminus to the terminus of a randomly selected path by adding a new breakpoint edge. Continue the process of joining paths until all paths with observed breakpoint edges are consumed and finishing with the path containing the end node. With the continuous path as input, GRIMM (12) finds a sequence of inversions transforming the original segment order into the rearranged chromosome. Note that once the breakpoint graph is created, all rearrangements impact only the breakpoint junctions between segments, and breakpoint reuse is allowed (13).

Deleted segments are found in isolated connected components comprising of exactly two nodes and one segment edge. In Figure 1b, there are four deleted segments. The last possibility for connected components in the graph are cycles, which represent duplicated segments. For example, a simple cycle with a breakpoint edge connecting the head of a segment to its tail is a tandem duplication. To determine the order of rearrangements to create the observed chromosome, deletions are randomly placed in the inversion order and duplications are introduced last to preserve low copy number states.

## RESULTS

### Simulating progressive rearrangements

**Figure 3.**
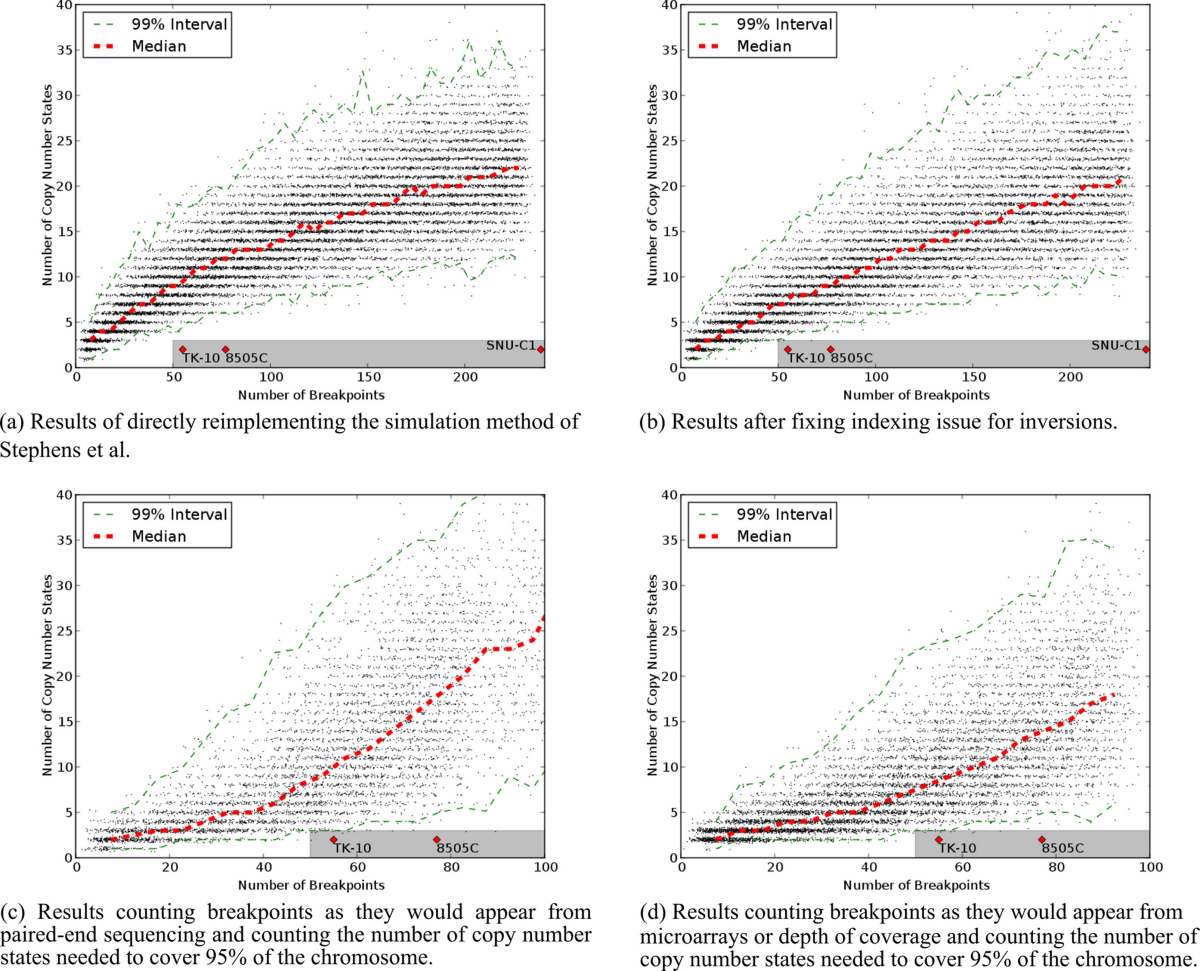
Charts of number of breakpoints versus number of copy number states for simulated chromosomes. The shaded gray area indicates the boundaries of the footprint of chromothripsis proposed by Stephens *et al.* The cell lines with number of breakpoints and copy number states as described by Stephens *et al.* are plotted as red points. The red dashed line shows the median number of copy number states for given numbers of breakpoints. The green dashed lines show an interval of copy number states that contains 99% of observations. (**a**) Results of directly reimplementing the simulation method of Stephens *et al.* (**b**) Results after fixing indexing issue for inversions. (**c**) Results counting breakpoints as they would appear from PES and counting the number of copy number states needed to cover 95% of the chromosome. (**d**) Results counting breakpoints as they would appear from microarrays or depth of coverage and counting the number of copy number states needed to cover 95% of the chromosome.

Stephens *et al.* graciously shared the code they used to produce their results. We have reimplemented the method, applied it to chromosome 15 of SNU-C1 and replicated their results (Figure 3a). The general trend, consistent with Stephens’ result, is that the number of unique copy number states increases with the number of breakpoints. A chromosome with 239 breakpoints and only two copy number states falls well outside of what was produced by the progressive simulation, and this is a key piece of evidence that chromosome 15 of SNU-C1 is the result of chromothripsis rather than progressive rearrangement. Moreover, based on the chart, it appears that a chromosome with at most three copy number states and more than 50, or perhaps even 20, breakpoints also falls outside of what can be achieved by progressive rearrangement.

### Chromothripsis footprint criteria depend on subtle simulation implementation details

The above result is more meaningful if it is robust to changes in the implementation of the simulation. In this section, we alter the simulation in various ways to determine if the proposed footprint of chromothripsis remains valid when assumptions about progressive rearrangement are changed.

The first change we made to the simulation was a correction of a logic error that caused some simulated inversions to behave like duplications. The details are in the Supplementary data, but the net effect was that some operations that ought to have preserved existing copy numbers instead introduced up to two new copy number states to the chromosome. When we corrected this, the chart of copy number states and breakpoints shifted down (Figure 3b). This change in result does not affect inferences about chromosome 15 of SNU-C1, since 239 breakpoints and only two copy number states are still well outside of the simulated results. But, the simulated chromosomes now begin to encroach upon the chromothripsis region of the graph. For example, the new simulation produced a chromosome with 67 breakpoints and only 3 copy number states, which is consistent with the footprint of chromothripsis even though the chromosome was rearranged progressively.

The next alteration was to the counting of breakpoints and copy number states. Thus far, we have been imprecise about the meaning of the breakpoint values on the x-axes of our charts. This imprecision is also found in the literature, but there are in fact multiple ways to count breakpoints on a chromosome. One way is to count the number of times an abnormal adjacency appears. For example in the chromosome in Figure 2f, moving from left to right we find eight such adjacencies: a(e), e(-c), (-c)(-g), (-g)(-h), (-f)d, hg, g(-i) and (-h)j. This counting method was used in Figure 3a and b. Another way to count breakpoints is to consider how the breakpoints would be reported by a PES experiment (14). This is similar to the previous method, except that if an abnormal adjacency appears in the chromosome multiple times because of duplications, it will only appear once in the sequencing results. So referring back to Figure 2f, the adjacencies hg and (-g)(-h) would count as one adjacency, even though they appear twice on the chromosome. A third way to count breakpoints is to consider how they will appear in a microarray or depth of coverage experiment (15). This method counts breakpoints where copy number changes. The copy numbers in the chromosome in Figure 2e from left to right are 1,0,1,2,4,3,1. So, copy number changes six times.

There are also multiple ways to count the number of copy number states in a chromosome. The first we can call ‘strict’. With this method, we simply count the number of copy number states observed in the chromosome, regardless of how much of the chromosome is covered by any copy number state. In Figure 2f, there are five copy states observed, zero through four. Another method, which we will call ‘relaxed’, counts how many copy states are needed to cover some fraction of the chromosome. If we use the fraction 90%, then the relaxed number of copy states in the chromosome above is four because we can cover 90 bases using only four copy number states. Relaxed counting of copy states can be appropriate for identifying chromothripsis because it allows us to find chromosomes that are dominated by two or three copy number states but may have some small regions with other copy numbers because of subsequent alterations or experimental error.

The simulation by Stephens *et al.* used strict copy number state counting and the first breakpoint counting method, counting every unexpected adjacency even if duplicated. In contrast, the breakpoints observed in chromosome 15 of SNU-C1 come from PES, and the copy number state count of two was arrived at using relaxed counting. Microarray results show that the chromosome has six copy number states using strict counting (16).

We modified the simulation to use relaxed copy state counting that found how many copy number states were needed to cover 95% of the simulated chromosome. When this was combined with PES breakpoint counting, it produced the results in Figure 3c; when combined with microarray breakpoint counting, it produced Figure 3d. Because of the changes in breakpoint counting, the simulations could no longer quickly produce chromosomes with over 100 breakpoints. Both simulations also showed a continuation of the trend seen in Figure 3b with a narrowing separation between the simulated chromosomes and chromosomes bearing the footprint of chromothripsis. For example, of the 414 chromosomes in Figure 3c with between 50 and 55 breakpoints, 16 (3.9%) were dominated by three or two copy number states. This suggests that in a screen of many chromosomes, the proposed footprint of chromothripsis may produce false discoveries.

**Figure 4.**
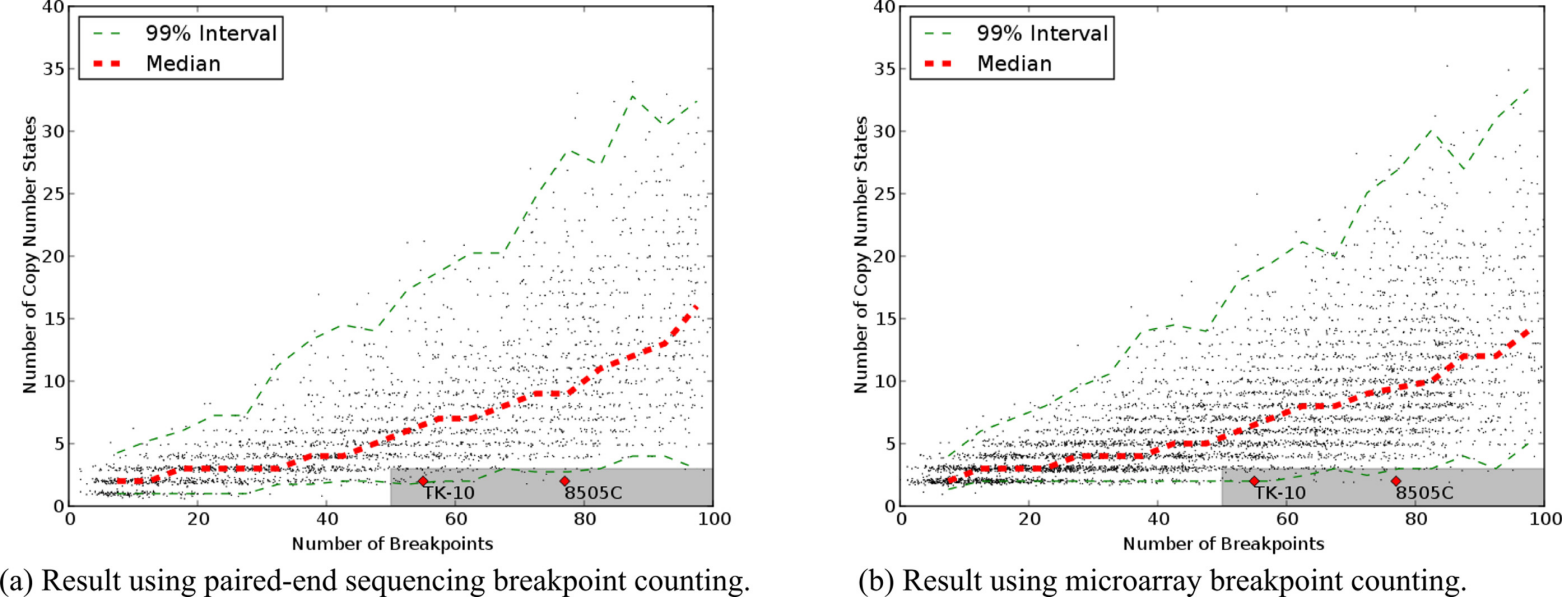
Charts of breakpoints versus copy number states for simulations with an overrepresentation of inversions. (**a**) Result using PES breakpoint counting. (**b**) Result using microarray breakpoint counting.

Finally, we altered the way the simulation chooses breakpoints to introduce into the chromosome. In the original simulation, breakpoints were chosen uniformly randomly without replacement, so each remaining breakpoint had an equal chance of being introduced at each step. This may not correspond to biological reality as there may be some preference for particular kinds of rearrangements. Specifically, a preference for inversions over other rearrangement types could lead to chromosomes with many breakpoints but few copy number states. To test this, we changed the simulation so that inversions were twice as likely to be chosen at each step compared to deletions or duplications. The results are in Figure 4a and b, using PES and microarray breakpoint counting, respectively. These results have many simulated chromosomes bearing the footprint of chromothripsis. The large fraction of chromosomes with many breakpoints and few copy number states (Table 2) indicates that some chromosomes that appear to have undergone chromothripsis could also have been produced by progressive rearrangement that favors inversions.

**Table 2.** Fraction of chromosomes in Figure 4a with few copy number states for given breakpoint counts

Breakpoint range	Fraction of chromosomes with two or three copy number states
50–59	12.6%
60–69	7.4%
70–79	2.6%
80–89	0.8%
90–99	0.6%

The results in this section suggest that a more conservative threshold should be used to identify chromothripsis in order to avoid false discoveries. If the minimum number of breakpoints were set at 100 rather than 50, much of the risk of false discovery we have demonstrated above would be diminished. However, this threshold would also decrease the estimate of the prevalence of chromothripsis. When Stephens *et al.* screened 746 cancer cell line copy number profiles for chromosomes with over 50 breakpoints and at most three copy number states, they found chromosomes from 18 cell lines that met these criteria. With a threshold of 100 breakpoints, the number of cell lines drops to 3. Based on this analysis, the true prevalence of chromothripsis may be less than .5% rather than the original estimate of 2–3%.

### Simulation method does not distinguish between progressive rearrangement and chromothripsis

In the previous section, we discussed implementation details of simulations of progressive rearrangements. We now turn our attention to the question of whether such simulations can provide reliable evidence for chromothripsis at all. In order for an experiment to provide information about a hypothesis, it has to produce different results when the hypothesis is true than when it is false. In order for simulations to demonstrate whether a chromosome could have been rearranged progressively, the simulations should produce different results for progressively rearranged chromosomes and chromosomes that have undergone chromothripsis.

The footprint of chromothripsis, many breakpoints with few unique copy states, is unlikely to appear in a chromosome rearranged by progressive and overlapping tandem duplications. However, it may appear in a chromosome rearranged by progressive inversions and deletions. We simulated such a chromosome with only inversions and deletions. The resulting breakpoints and copy numbers are shown in Supplementary Figure S1. The chromosome had 237 breakpoints and only two copy number states, zero and one. Even though only two kinds of rearrangements were used, the chromosome shows the same complex rearrangement pattern seen in chromosomes that have putatively undergone chromothripsis.

**Figure 5.**
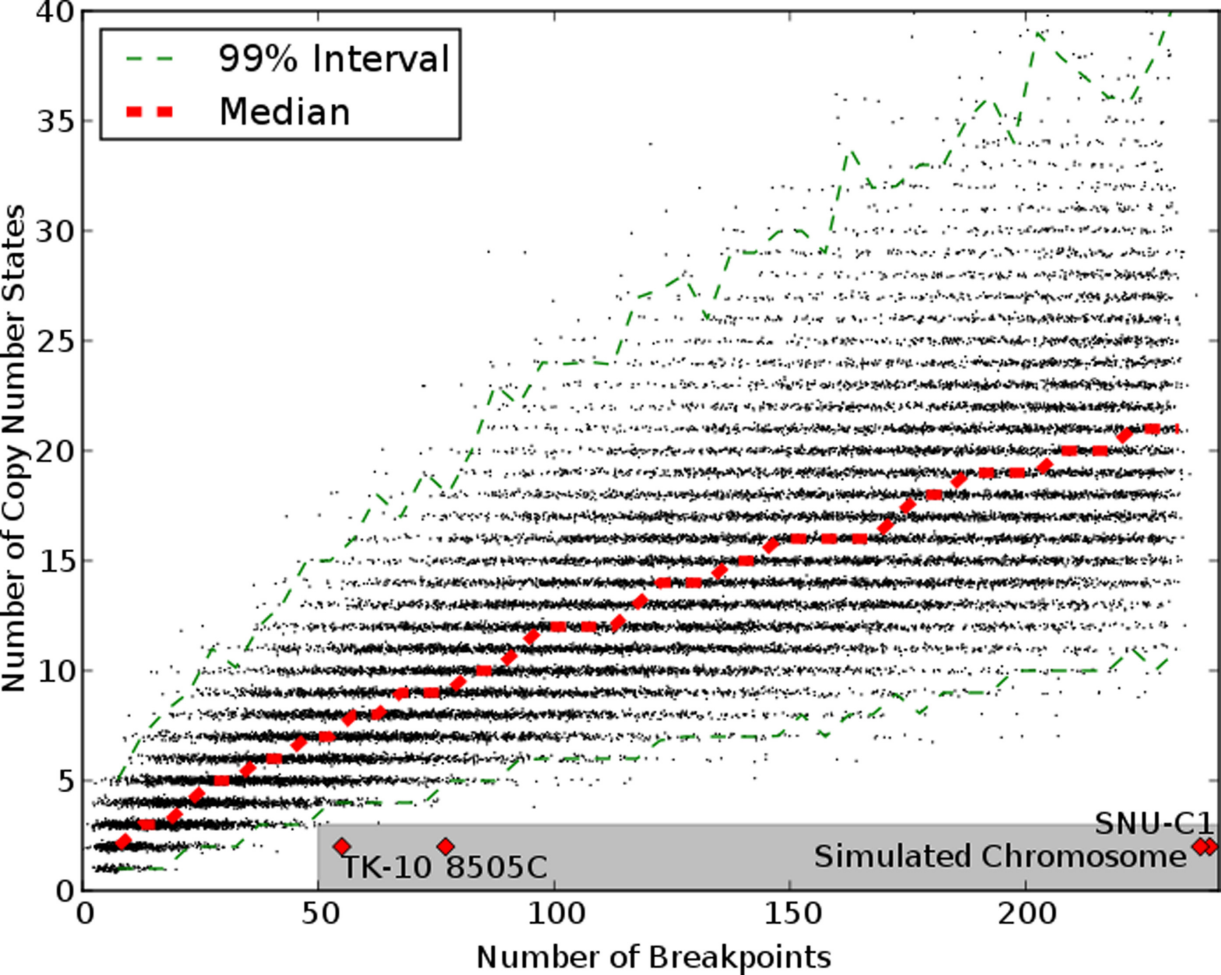
Counts of breakpoints and copy number states from a simulation based on the breakpoints from simulated chromosome in Supplementary Figure S1. The breakpoints and copy number states of the simulated chromosome are indicated on the chart.

We then applied the simulation method to the breakpoints of this chromosome and recorded the results as we did in Figure 3b. The resulting distribution of breakpoints and copy number states in Figure 5 is not different from Figure 3b even though we know the chromosome was rearranged progressively. This result casts doubt on the usefulness of the simulation method to detect chromothripsis. Rather than distinguishing between chromosomes that shattered and chromosomes that were rearranged progressively, it always reports that chromosomes with many complex rearrangements and few copy number states are the product of chromothripsis even when they are not.

### Plausible progressive rearrangement schemes exist for chromosomes bearing footprint of chromothripsis

Thus far, we have discussed in general whether some chromosomes that appear to be the product of chromothripsis may actually have been progressively rearranged. We now move from the general to the specific to see if we can find series of progressive rearrangements that explain particular chromosomes that bear the footprint of chromothripsis. Stephens *et al.* singled out three chromosomes from three different cell lines for extensive sequencing and analysis: chromosome 5 from TK10, chromosome 9 from 8505C and chromosome 15 from SNU-C1. These chromosomes had 55, 77 and 239 breakpoints respectively and oscillated between two copy number states. We developed a method that explains these breakpoints and copy number states using only progressive rearrangements.

As discussed above, one way to ensure that a chromosome has no more than two copy states is to only rearrange it by deletions and inversions. The problem of explaining genomic rearrangements using inversions alone, also known as the ‘Sorting by reversals’ problem, was solved by the Hannenhalli–Pevzner theory (17), and implemented in the tool GRIMM (12). In this problem, the input is a pair of chromosomes with identical, but highly rearranged genomic content. The output is a sequence of inversions that transforms one chromosome to the other. For each of the three chromothripsis chromosomes, we identified arrangements of chromosomal segments that would yield the observed breakpoints using a graph traversal technique. In addition, some of the breakpoints support missing chromosomal segments. These missing segments were removed by progressively introducing deletions. Also, breakpoints supporting tandem duplications are resolved last to ensure the fewest copy number states are observed. GRIMM then revealed inversions that would convert the unrearranged chromosome into one with the observed breakpoints (see Materials and Methods).

**Figure 6.**
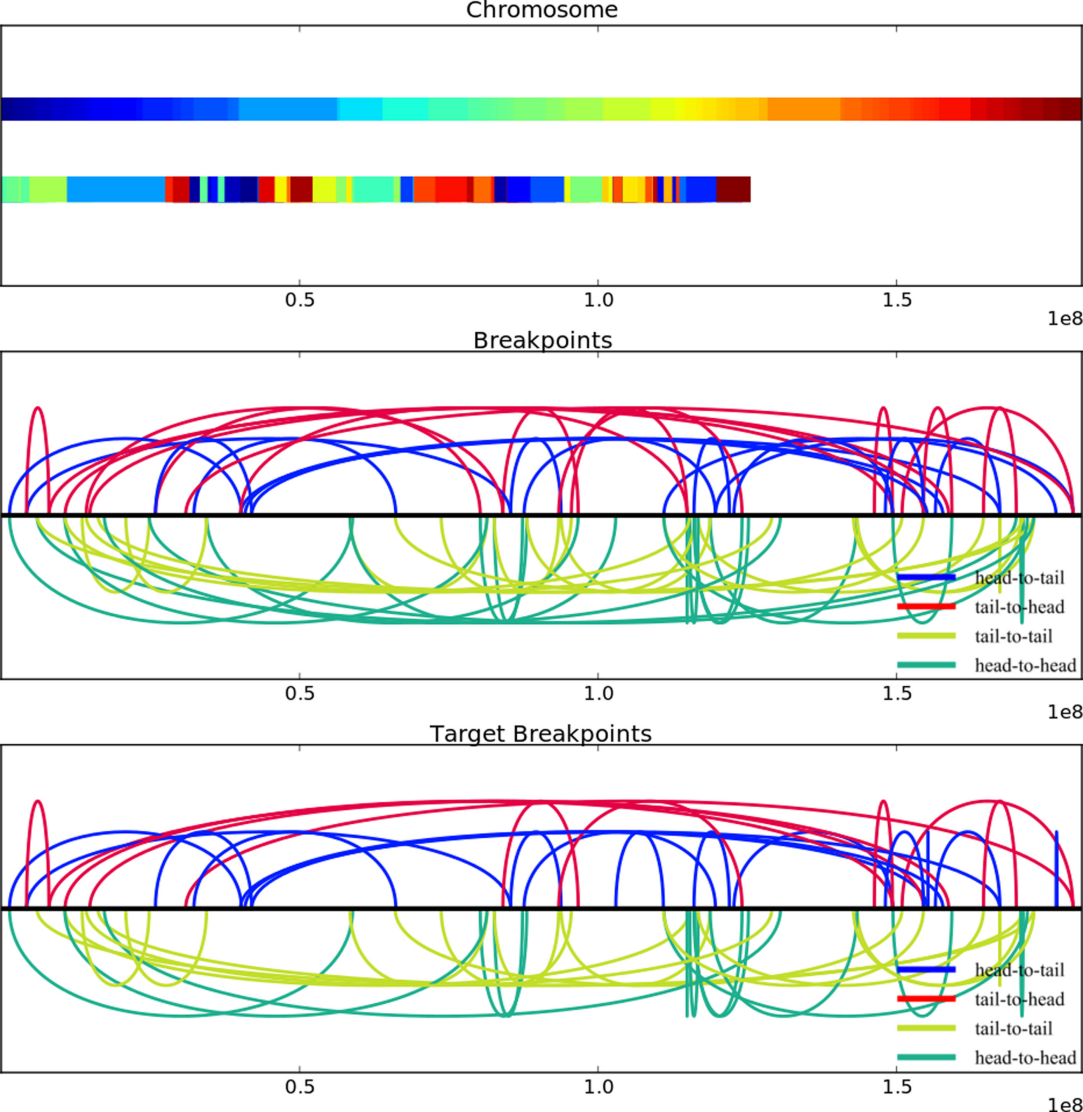
An illustration of the result of the series of inversions and deletions for chromosome 5 of TK10. The top panel broadly shows the ordering of segments after rearrangement. The upper color bar shows all segments of the unrearranged chromosome colored from blue to red. The lower color bar shows segments with the same coloring after rearrangement. Note that some segments have been deleted so the chromosome is shorter. The middle panel shows the breakpoints achieved by inversions and deletions, and the lower panel shows the observed breakpoints.

For each of the three chromosomes, we identified a sequence of inversions, deletions and tandem duplications that yielded 100% of the experimentally observed breakpoints as well as some additional breakpoints beyond what was observed (Table 3). Figure 6 illustrates the result for chromosome 5 from TK10. Animations of the series of rearrangements for each of the three chromosomes are in the Supplementary data.

**Table 3.** For each of the three chromothripsis chromosomes, the number of breakpoints observed experimentally, the number of unobserved breakpoints that were produced by the inversions and deletions and the number of progressive rearrangement events broken down by rearrangement type

Cell line	Experimental breakpoints	New breakpoints	Progressive inversions	Progressive deletions	Progressive tandem duplications
TK10	55	12	63	42	3
8505C	77	21	91	56	1
SNU-C1	239	92	321	145	2

These series of progressive rearrangements raise potential alternative hypotheses for the complex breakpoints and oscillating copy number states in these chromosomes. Thus, while these chromosomes may have indeed undergone chromothripsis, the observations can also be explained using progressive rearrangements alone.

### The alternative explanation

Above, we have shown that simulations and the observed pattern of low copy number state count and high number of breakpoints clearly cannot distinguish chromothripsis from progressive rearrangements that favor inversions and deletions. However, we do not claim that a particular scheme of simple inversions and simple deletions causes the observed phenomenon. Inversions are the simplest generalization of a larger class of balanced rearrangements, which include translocations and rearrangements with *multiple* breakpoints. Specifically, define a *k*-break rearrangement, as an operation that rearranges *k* − 1 distinct segments of a chromosome creating *k* breakpoints. By this definition, an inversion is a specific type of 2-break rearrangement, while transpositions (and inverted transpositions) are examples of 3-break rearrangements. Consistent with genome rearrangement theory (11,18,19), a *k*-break rearrangement can be equivalently explained by a series of 2-break rearrangements (reviewed in Supplementary Section 3). Using the *k*-break definition, chromothripsis can be described as an *n*-break rearrangement allowing for deletions. Our results show that a signature consisting only of breakpoints cannot distinguish between progressive 2-break rearrangements and deletions from a one-off *n*-break rearrangement with deletions (chromothripsis). Since 2-break rearrangements decompose *k*-break rearrangements, progressive combinations of *k*-break rearrangements for 2 ≤ *k* < *n* are equally plausible explanations for the observed breakpoints and copy number states. As each of these alternative scenarios provides a different number of rearrangement events, the number of rearrangement events cannot be accurately estimated using only breakpoint and copy number data.

## DISCUSSION

It is notoriously difficult to make sense of many cancer genomes due to the complexity of rearrangements. The proposal of the chromothripsis hypothesis was an important step forward as a possible mechanism for the creation of this complexity. Careful investigation of the phenomenon may deepen knowledge of structural variation in cancers.

At the same time, the proposal of ‘shattering and subsequent reassembly’ of a chromosome in a single catastrophic event is truly extraordinary. The invocation of chromothripsis to explain molecular data from cancer samples must be done with great circumspection, and caution, even. The case for chromothripsis rests on the argument that there are some patterns of variation that progressive rearrangement cannot achieve. But in this paper, we have shown that progressive rearrangements can indeed achieve patterns that, at first glance, would seem quite unlikely. The primary evidence supporting chromothripsis (1) is (1) high breakpoint count and low copy number states. We demonstrated that this footprint of chromothripsis, in fact, includes chromosomes rearranged progressively, that simulations might always rule out progressive rearrangement regardless of how the chromosome truly evolved and that it is possible to find progressive rearrangements that explain chromosomes that appear to be exemplars of chromothripsis.

Additional criteria used to argue for a chromothripsis event are: (2) clustering of breakpoint locations, (3) randomness of fragment joins, (4) rearrangements affecting a single haplotype, (5) interspersed loss and retention of heterozygosity and (6) ability to walk the derivative chromosome (1,10). However, these new criteria do not preclude chromosome formation via progressive rearrangements. For example, progressive rearrangements may produce the same pattern of (2) clustered breakpoint locations and (3) randomess of fragment joins. In our progressive rearrangement simulations, breakpoints were sampled from the exemplar chromosome 15 of SNU-C1, which has an identical distribution of breakpoint locations and breakpoint orientations. A recent review (10) reported patterns (4), (5) and (6), but did not provide quantitative analysis of these patterns against the few chromosomes proposed to have undergone chromothripsis. Pattern (4) suggests that breakpoints falling on one chromosome versus two homologous chromosomes is an indication of chromothripsis. Again, the pattern is indiscriminate since rearrangements may still progressively fall on a single chromosome. Additionally, patterns (5) and (6) are features specifically of rearrangements appearing on the same chromosome. (5) Interspersed loss and retention of heterozygosity occurs when segments are deleted from only one of the homologous chromosomes. As the other homologous chromosome is intact, segments that are deleted appear to have loss of heterozygosity and remaining segments retain heterozygosity. The property of (6) walking the derivative chromosome states that a set of chromothripsis breakpoints when projected onto a reference chromosome will allow for an unambiguous walk from one end of the reference chromosome to the other end of the chromosome traversing all the observed breakpoints. If both homologous chromosomes have rearrangements and since it is not known which breakpoints arise from which chromosome, finding an unambiguous walk is typically not possible. Along the reference there would be two walks and for each walk, inference would have to be made on which breakpoints to follow. However, if only one of the homologous chromosomes is rearranged, a single unambiguous walk is possible using all the breakpoints supporting the single rearranged chromosome. In our analysis, like Stephens *et al.*, we assume progressive rearrangements are occurring on the same chromosome and likewise the proposed patterns (4), (5) and (6) are inherently reproduced with progressive rearrangements in our simulations and plausible explanations of documented chromothripsis chromosomes.

In balance, our results suggest it is difficult to point to statistical evidence that predicts chromothripsis while excluding other possibilities. In this manuscript, we do not delve into biological explanations of the observed rearrangement patterns. For example, if rearrangements accumulate progressively, how could they be limited to a single chromosome. Is not a one-off catastrophic chromothripsis event a better explanation than some ‘memory’ that causes the same chromosome (or a few chromosomes) to be dramatically rearranged over time? However, there is evidence that chromosomal lesions might make a chromosome more susceptible to mutations. An example is provided by the breakage-fusion-bridge mechanism where the loss of a telomere and resulting instability leads to progressive cycles of rearrangements. Indeed, Sorzano *et al.* propose the breakage-fusion-bridge and similar progressive mechanisms show patterns similar to chromothripsis (20). Chiang *et al.* (21) showed transgene integration in germline cells makes a chromosome more susceptible to rearrangements and the resulting complex rearrangements have chromothripsis like patterns. While they suggest the rearrangements appear in a one-off event, we note that there are still numerous cell divisions between pronucleus injection of exogenous DNA and harvesting of DNA for sequencing. Based on this, we cannot rule out that rearrangements accrued over a few cell divisions. The rearrangements do not need to keep occurring in all cell divisions as well. For example, breakage-fusion-bridge cycles occur across distinct cell divisions, but eventually lead to a stable rearranged chromosome. Similarly, Liu *et al.* ((22), Table 1) provide numerous examples of complex rearrangements occurring on a single chromosome. They suggest that the results are best explained not as chromothripsis, but due to a ‘chromoanasynthesis’, as they involve errors in replicative mechanisms and show duplications and triplications not seen in chromothripsis. They provide the example of a patient with 18 copy number changes including a 5.5 Mbp triplicated and inverted segment. Breakpoint analysis revealed insertions of long (1.5Kbp) novel sequences at breakpoints, which might provide a template switch during the replicative process (22). Once we admit these possibilities, however, the difference between one-off and progressive events becomes harder to measure.

We find purely statistical evidence cannot distinguish between one-off and progressive events (see also (23,20)). Other authors have noted that ‘chromothripsis like events’ cannot be distinguished from other complex genomic rearrangements (24). While we use inversions and deletions to explain the chromothripsis patterns, we do not claim a particular scheme of simple inversions and simple deletions causes the phenomenon. Inversions are the simplest generalization of a larger class of balanced rearrangements, which include translocations and rearrangements with *multiple* breakpoints (see Supplementary Section 3). In other words, the chromothripsis pattern could also be explained by progressive steps of balanced rearrangements like translocations. Lastly, the appearance of balanced rearrangements and deletions is a plausible scenario for cancer. Wang *et al.* (25) analyzed five T-ALL samples and observed 31 interchromosomal translocations, 19 intrachromosomal translocations, 1 inversions, 22 deletions and 16 insertions. While they did not describe copy number changes, the number of breakpoints from possible copy neutral events is high (31 + 19 + 1 × 2 = 52 breakpoints). Thus, it is reasonable to find a few extreme cancer chromosomes having a higher number of balanced rearrangements and deletions.

## CONCLUSION

These results do not foreclose upon the chromothripsis hypothesis, of course. But, they do underscore difficulty of making inferences about mechanisms in cancer. Indeed, there is no doubt that some of the cancer genomes have undergone extensive rearrangements. At the same time, the evidence is limited for the claim that a single catastrophic event joined shattered DNA together, and requires additional investigation before it can be accepted as established fact. For now, chromosomes with many breakpoints should be labeled as having undergone complex genome rearrangements rather than implying a shattering mechanism by chromothripsis. Future advances in single-cell sequencing and haplotype resolved genome assembly might shed light on the mechanisms underlying complex rearrangements.

## SUPPLEMENTARY DATA

Supplementary Data are available at NAR Online, including [26,27].

SUPPLEMENTARY DATA

## References

[1] Stephens P.J., Greenman C.D., Fu B., Yang F., Bignell G.R., Mudie L.J., Pleasance E.D., Lau K.W., Beare D., Stebbings L.A. (2011). Massive genomic rearrangement acquired in a single catastrophic event during cancer development. Cell.

[2] Magrangeas F., Avet-Loiseau H., Munshi N.C., Minvielle S. (2011). Chromothripsis identifies a rare and aggressive entity among newly diagnosed multiple myeloma patients. Blood.

[3] Rausch T., Jones D.T., Zapatka M., Stutz A.M., Zichner T., Weischenfeldt J., Jager N., Remke M., Shih D., Northcott P.A. (2012). Genome sequencing of pediatric medulloblastoma links catastrophic DNA rearrangements with TP53 mutations. Cell.

[4] Northcott P.A., Shih D.J., Peacock J., Garzia L., Morrissy A.S., Zichner T., Stutz A.M., Korshunov A., Reimand J., Schumacher S.E. (2012). Subgroup-specific structural variation across 1,000 medulloblastoma genomes. Nature.

[5] Molenaar J.J., Koster J., Zwijnenburg D.A., van Sluis P., Valentijn L.J., van der Ploeg I., Hamdi M., van Nes J., Westerman B.A., van Arkel J. (2012). Sequencing of neuroblastoma identifies chromothripsis and defects in neuritogenesis genes. Nature.

[6] Kloosterman W.P., Hoogstraat M., Paling O., Tavakoli-Yaraki M., Renkens I., Vermaat J.S., van Roosmalen M.J., van Lieshout S., Nijman I.J., Roessingh W. (2011). Chromothripsis is a common mechanism driving genomic rearrangements in primary and metastatic colorectal cancer. Genome Biol..

[7] Kloosterman W.P., Guryev V., van Roosmalen M., Duran K.J., de Bruijn E., Bakker S.C., Letteboer T., van Nesselrooij B., Hochstenbach R., Poot M. (2011). Chromothripsis as a mechanism driving complex de novo structural rearrangements in the germline. Hum. Mol. Genet..

[8] Chiang C., Jacobsen J.C., Ernst C., Hanscom C., Heilbut A., Blumenthal I., Mills R.E., Kirby A., Lindgren A.M., Rudiger S.R. (2012). Complex reorganization and predominant non-homologous repair following chromosomal breakage in karyotypically balanced germline rearrangements and transgenic integration. Nat. Genet..

[9] Patel A., Hixson P., Bi W., Borgan C., Coyle M., Freppon D., Vo D., O'Hare J.T., Luke P., Chang C.-C. (2011). Is it time for array CGH to be the first line test for detection of chromosome abnormalities in hematological disorders-example multiple myeloma. Blood.

[10] Korbel J.O., Campbell P.J. (2013). Criteria for inference of chromothripsis in cancer genomes. Cell.

[11] Bafna V., Pevzner P.A. (1996). Genome rearrangements and sorting by reversals. SIAM J. Comput..

[12] Tesler G. (2002). Efficient algorithms for multichromosomal genome rearrangements. J. Comput. Syst. Sci..

[13] Alekseyev M.A., Pevzner P.A. (2007). Are there rearrangement hotspots in the human genome?.

[14] Tuzun E., Sharp A.J., Bailey J.A., Kaul R., Morrison V.A., Pertz L.M., Haugen E., Hayden H., Albertson D., Pinkel D. (2005). Fine-scale structural variation of the human genome. Nat. Genet..

[15] Yoon S., Xuan Z., Makarov V., Ye K., Sebat J. (2009). Sensitive and accurate detection of copy number variants using read depth of coverage. Genome Res..

[16] Greenman C.D., Bignell G., Butler A., Edkins S., Hinton J., Beare D., Swamy S., Santarius T., Chen L., Widaa S. (2010). PICNIC: an algorithm to predict absolute allelic copy number variation with microarray cancer data. Biostatistics.

[17] Hannenhalli S., Pevzner P. (1995). Transforming Cabbage into Turnip (polynomial algorithm for sorting signed permutations by reversals). *Journal of the ACM*.

[18] Bafna V., Pevzner P.A. (1998). Sorting by transpositions. SIAM J. Discrete Math..

[19] Alekseyev M.A., Pevzner P.A. (2008). Multi-break rearrangements and chromosomal evolution. Theor. Comput. Sci..

[20] Sánchez Sorzano C.O., Pascual-Montano A., Sánchez de Diego A., Martínez-A C., van Wely K.H. (2013). Chromothripsis: Breakage-fusion-bridge over and over again. Cell Cycle.

[21] Chiang C., Jacobsen J.C., Ernst C., Hanscom C., Heilbut A., Blumenthal I., Mills R.E., Kirby A., Lindgren A.M., Rudiger S.R. (2012). Complex reorganization and predominant non-homologous repair following chromosomal breakage in karyotypically balanced germline rearrangements and transgenic integration. Nat. Genet..

[22] Liu P., Erez A., Nagamani S. C.S., Dhar S.U., Kołodziejska K.E., Dharmadhikari A.V., Cooper M.L., Wiszniewska J., Zhang F., Withers M.A. (2011). Chromosome catastrophes involve replication mechanisms generating complex genomic rearrangements. Cell.

[23] Righolt C., Mai S. (2012). Shattered and stitched chromosomeschromothripsis and chromoanasynthesismanifestations of a new chromosome crisis?. Genes Chromosomes Cancer.

[24] Malhotra A., Lindberg M., Faust G.G., Leibowitz M.L., Clark R.A., Layer R.M., Quinlan A.R., Hall I.M. (2013). Breakpoint profiling of 64 cancer genomes reveals numerous complex rearrangements spawned by homology-independent mechanisms. Genome Res..

[25] Wang J., Mullighan C.G., Easton J., Roberts S., Heatley S.L., Ma J., Rusch M.C., Chen K., Harris C.C., Ding L. (2011). CREST maps somatic structural variation in cancer genomes with base-pair resolution. Nat. Methods.

[26] McPherson A., Wu C., Wyatt A.W., Shah S., Collins C., Sahinalp S.C. (2012). nFuse: Discovery of complex genomic rearrangements in cancer using high-throughput sequencing. Genome Res..

[27] Kececioglu J., Sankoff D. (1995). Exact and approximation algorithms for sorting by reversals, with application to genome rearrangement. Algorithmica.

