# Supplementary material for: The elusive evidence for chromothripsis
Source: Nucleic Acids Res. 2014 Jun 17;42(13):8231–42. doi: 10.1093/nar/gku525 (PMC4117757; doi:10.1093/nar/gku525)
Supplement: SUPPLEMENTARY DATA [file supp_42_13_8231__index.html]

The elusive evidence for chromothripsis — SUPPLEMENTARY DATA 

# The elusive evidence for chromothripsis

## SUPPLEMENTARY DATA

**Files in this Data Supplement:**

- Supplementary Data 4
- Supplementary Data 1
- Supplementary Data 2
- Supplementary Data 3
