# Supplementary material for: The elusive evidence for chromothripsis
Source: Nucleic Acids Res. 2014 Jun 17;42(13):8231–42. doi: 10.1093/nar/gku525 (PMC4117757; doi:10.1093/nar/gku525)
Supplement: SUPPLEMENTARY DATA [file supp_gku525_nar-00163-n-2014-File009.pdf]

Supplement for "The elusive evidence for chromothripsis."

May 14, 2014

1 Indexing issue in the simulations from Stephens et al.

The simulations of progressive rearrangements were implemented in R by Stephens *et al.* The ordering of segments in the chromosome was maintained in a data frame called `curr.state`. Different types of rearrangements were effected by manipulating `curr.state`. A "head-to-head" inversion could be created with the following code:

```
curr.state <- curr.state[c(1:left.seg, right.seg:(left.seg+1),
   (right.seg+1):(dim(curr.state)[1])),]
```

Here, `left.seg` and `right.seg` refer to the positions of the segments that are the boundaries of the region being inverted. If `left.seg` $\leq$ `right.seg`, the code correctly creates an inversion in `curr.state`. However, because of the varying orientations of segments in the simulated chromosome, it may be the case that `left.seg` $>$ `right.seg`. If this is so, this code will triplicate [`right.seg+1`,`left.seg`] and will duplicate the segments at `right.seg` and `left.seg+1`. A similar issue exists for "tail to tail" inversions. This error causes the simulation to introduce two new copy number states when it should not introduce any.

2 Simulation of a chromosome simulated with progressive inversions and deletion

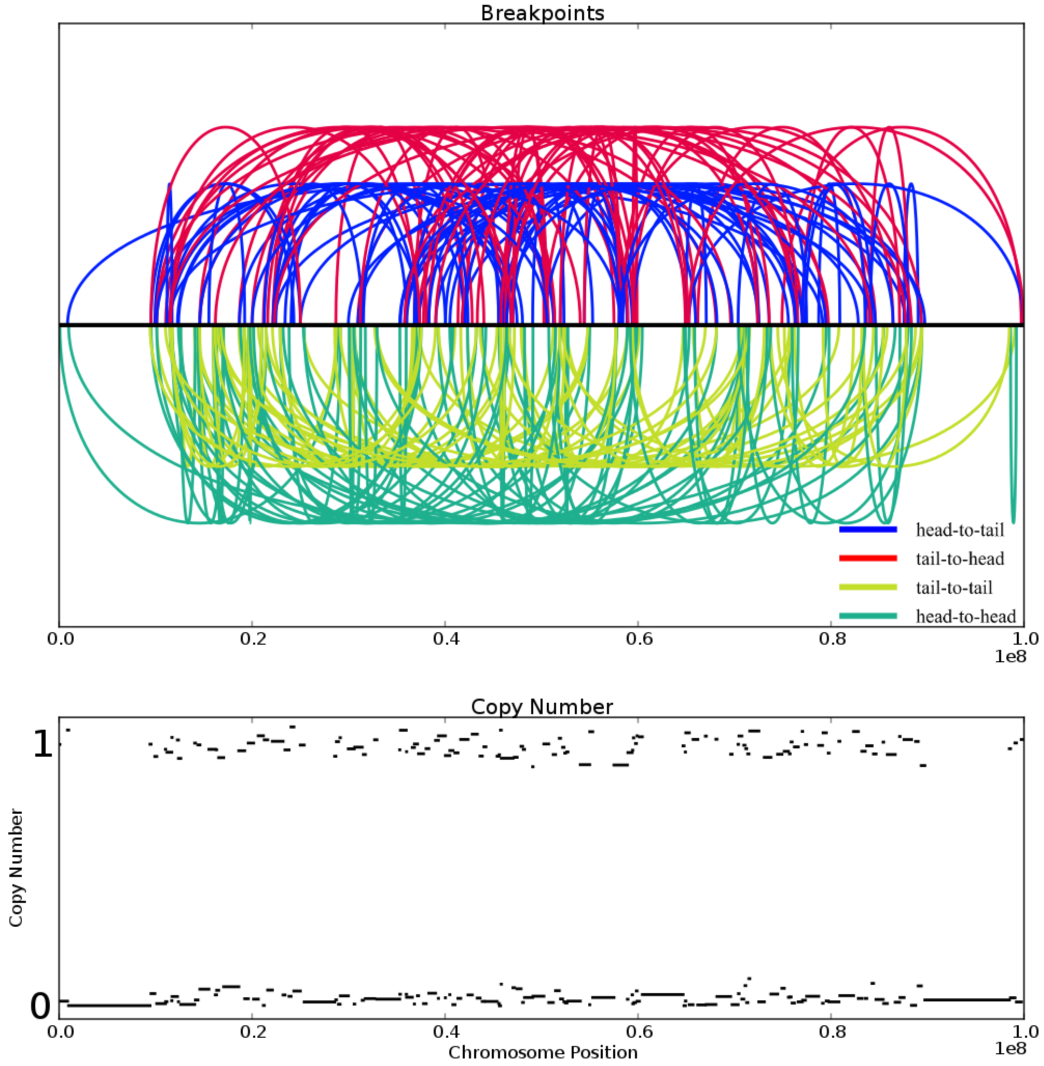

Figure 1: Breakpoints and copy number states of a chromosome simulated with progressive inversions and deletions. The number of breakpoints and copy number states is similar to SNU-C1. The breakpoints are colored according to orientation.

3 Equivalence of inversions, intra-chromosomal translocations, and inter-chromosomal translocations

Claim: *A genome that is rearranged by one or more copy number neutral events (i.e. intra-chromosomal translocations) can be equivalently explained by a sequence of inversions.*

Our manuscript suggests that a sequence of inversions suffice to explain the rearranged chromosome and copy number states of the cell-line SNU-C1, previously only explained by chromothripsis. This raises the natural question, *is a sequence of inversions a better biological explanation?* However, the question is misdirected. A more relevent question, *are the other biological explanations as plausible as chromothripsis?* We do not need to invoke inversions solely to explain the rearrangements. Here, we explain how any sequence of *copy-neutral* rearrangements can be equivalently explained by inversions. Conversely, a sequence of inversions can possibly be explained using a combination of copy-neutral rearrangements. To intuitively understand the equivalence, we consider three examples below.

- We show that an intra-chromosomal translocation is explained by a sequence of inversions.
- We define the concept of k-break rearrangements (also known as complex genome rearrangements), and show that both inversions and chromothripsis are special cases of k-break rearrangements.
- We extend these descriptions to *inter-chromosomal translocations*, and show that inter-chromosomal translocations can also be modeled as a sequence of inversions.

To reiterate, when we show that a complex rearrangement is modeled by a sequence of inversions, it is equivalent to saying that it is modeled by *some* sequence of copy neutral events. For more precise and thorough arguments of these ideas, see [11, 19, 18].

3.1 Intra-chromosomal translocation (transposition)

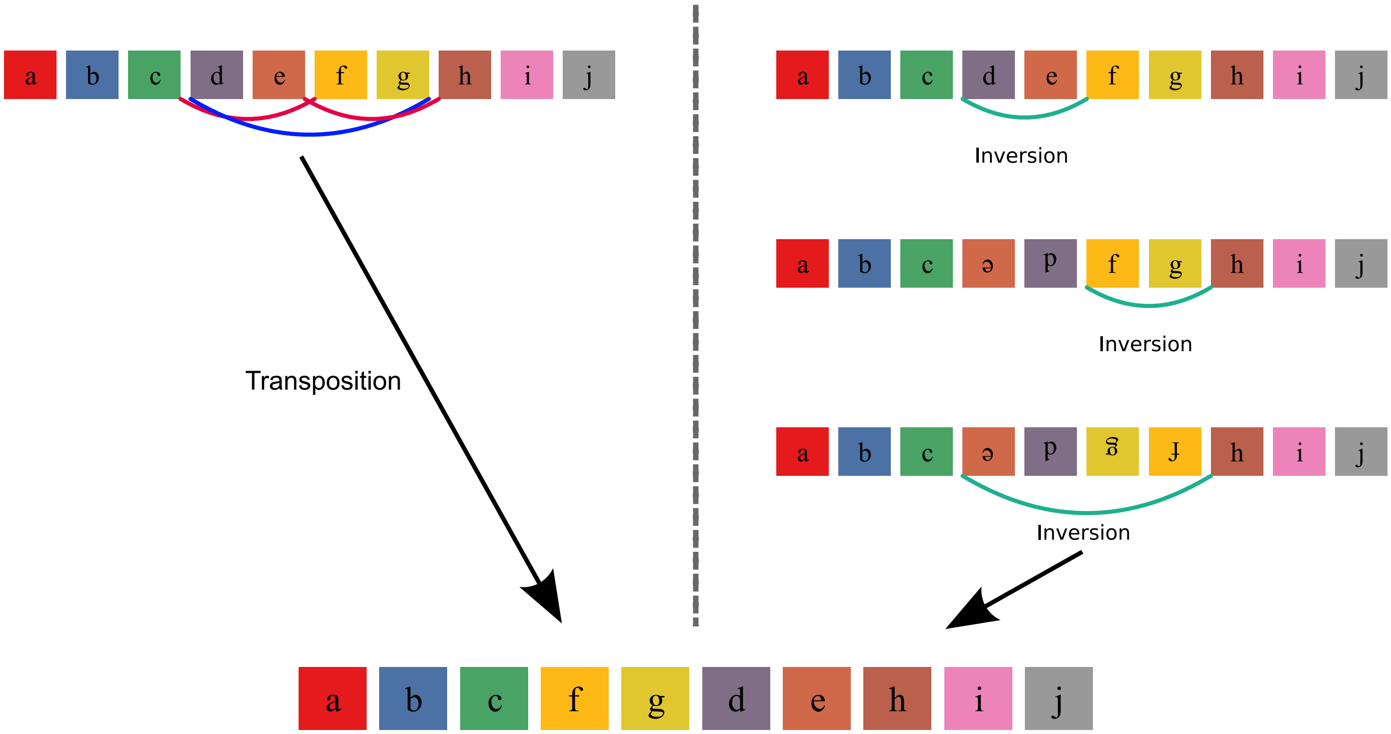

Figure 2: Transposition of 'de' into 'gh'.

The event of a DNA segment moving to a new position in the genome is referred to as transposition. An example of transposition is shown in Figure S2. In the example, the segment 'de' moves between 'g' and 'h', which rearranges "abc**de**fghij" to "abcfg**de**hij". The removal of 'de' creates two breaks in sequence, and the insertion breaks the sequence 'gh'. As a result, three breakpoint are observed, corresponding to the fusion of 'cf', 'gd', and 'eh'. While the singular

event is referred to as transposition, the breakpoints can also be explained by a sequence of three inversions, inversion of 'de', followed by inversion of 'fg', and finally inversion of '(-e)(-d)(-g)(-f)' (Figure S2). Contrarily, when we say that the rearrangements are explained by a sequence of inversions, we are only claiming that they are explained by some sequence of copy-neutral events.

3.2 Complex genome (or, k-break) rearrangements

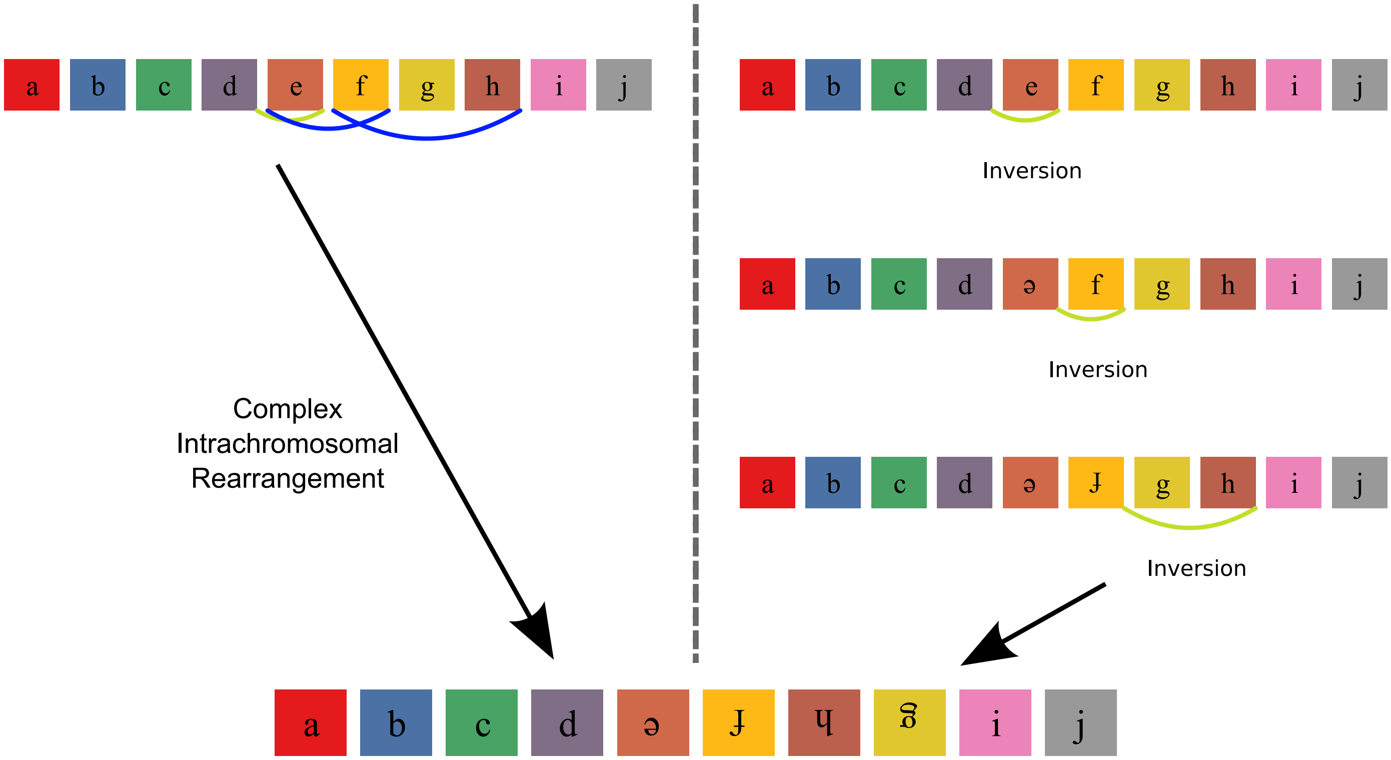

Figure 3: Complex genome rearrangement with three breakpoints. Poly-fusion event in chromosome 8 involving three genes discovered by [26].

It is an intuitive but misguided conclusion that rearrangements leave characteristic signatures of paired-end discordant mappings. Certainly for simple, single events, this is true, and we see characteristic signatures for deletions, inversions, etc., as reported by many authors [25]. However, when we look at complex rearrangements, the signatures are no longer predictive. Transposition is an intuitive example of a one step multi-break rearrangement, and we showed in the previous example that it could be equivalently explained by inversions. To generalize this, Alekseyev and Pevzner [19] developed the notion of k-break rearrangements and argued that as k increases the number of rearrangement events dropped dramatically. However, *given a rearranged genome, not much can be said about the type and number of rearrangements that created it.*

Here, we consider an empirical example. McPherson et al. [26] analyzed cancer RNA-seq data and found numerous examples of aberrant gene fusions that implied complex genome rearrangement events. In Figure S3, we illustrate a poly-fusion, observed on chromosome 8 by McPherson et al. Within the tumor chromosome, the authors observed a poly-fusion of genic and non-genic parts comprised of inverted PHF20L1, an inverted non-genic section between PHF20L1 and FAM49B, inverted FAM49B, and SAMD12. In Figure S3, chromosome 8 is represented by the sequence "abcdefghij" where segments represent genes as follows: 'd' corresponds to SAMD12, 'e' corresponds to FAM49B, 'f' corresponds to the non-genic section between PHF20L1 and FAM49B, and 'gh' is PHF20L1. The tumor chromosome 8 observed by McPherson et al. [26] in the block representation is "abcd(-e)(-f)(-h)(-g)ij". Since there was no evidence to support a particular DNA rearrangement mechanism, the authors refrained from suggesting how the complex event arose. We demonstrate two extreme scenarios for explaining the breakpoints observed in Figure S3. First, the breakpoints could be created in a single multi-break shuffle as shown in Figure S3. In the second scenario, the breakpoints could be explained by three sequential inversions, inversion of 'e', inversion of 'f', and inversion of 'gh'. *Both scenarios equally explain the rearranged tumor chromosome.* Thus, any claim that one or the other event is more likely must be supported by

other evidence. One could think of chromothripsis as another extreme scenario where *all* observed breaks are due to a single massive rearrangement. However, the statistical evidence from the rearranged genome cannot really distinguish chromothripsis from other sequences of copy neutral changes.

3.3 Inter-chromosomal rearrangements

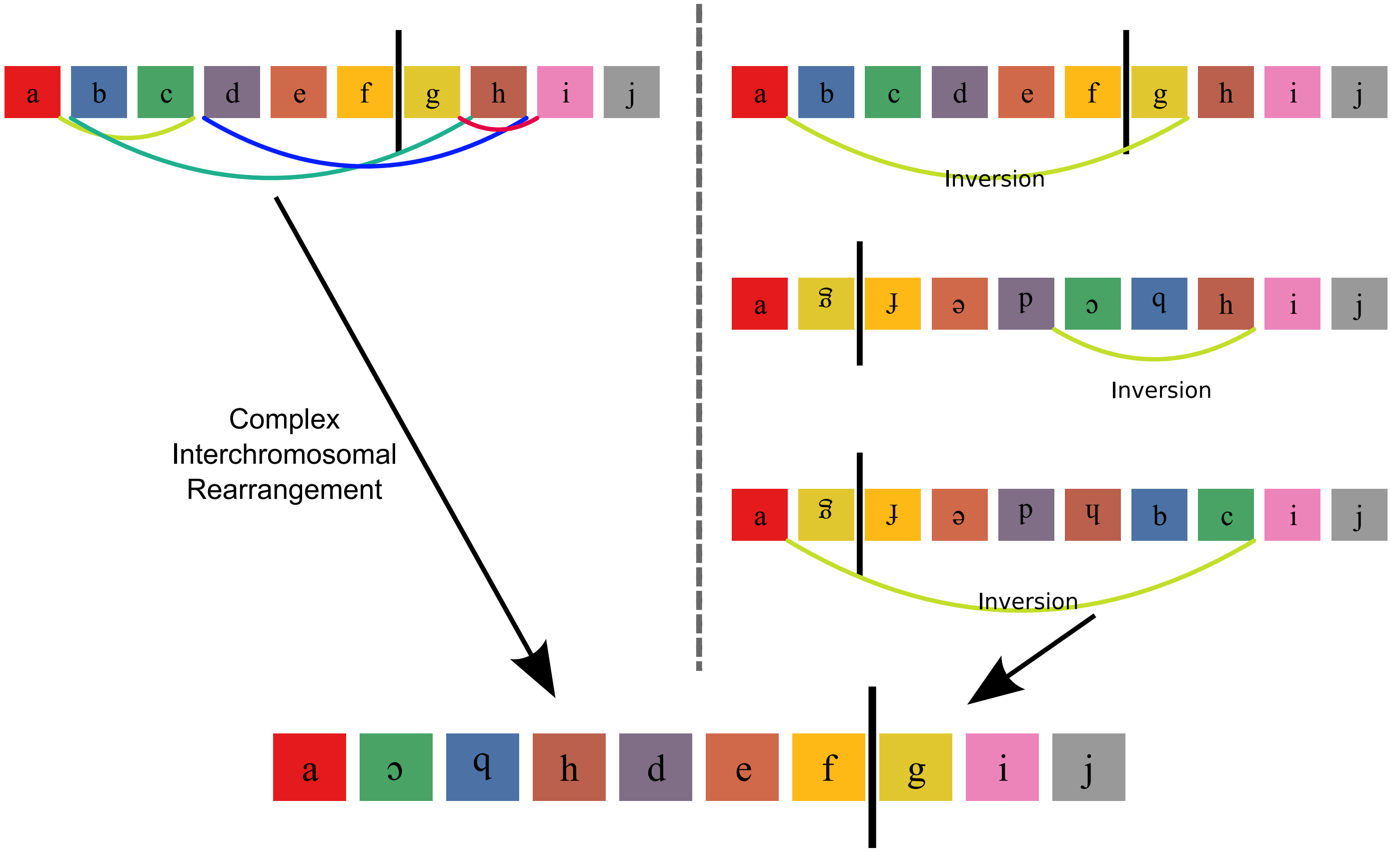

Figure 4: Complex genome rearrangement event across multiple chromosomes. “abcdef” represents chromosome 5 and “ghij” represents chromosome 8. The gene fusion event involving DNA segments from chromosome 5 and 8 was discovered by [26].

While chromothripsis was originally claimed to occur on a single chromosome, the mechanism has been been additionally claimed to affect loci clusters across multiple chromosomes [3, 5, 6, 8, 10]. Genome rearrangement theory extends inversions to even apply to rearrangements affecting multiple chromosomes, and once again, a progressive sequence of ‘inversions’ (a more generalized version) can be used to explain these breakpoints as well. Once again, we are only claiming that the statistical evidence cannot distinguish between a multitude of copy neutral events, or a single, catastrophic event.

McPherson et al. discovered another gene fusion where ZDHHC11 and RNF130 from chromosome 5 are fused together by a non-genic sequence from chromosome 8. In Figure S4, two extreme scenarios for rearrangement explain the breakpoints on the rearranged chromosomes. Here, chromosome 5 is labeled as “abcdef” and chromosome 8 is “ghij”. The ZDHHC11 and RNF130 genes are represented by segments ‘bc’ and ‘de’, respectively. The observed tumor chromosome 5 is “a(-c)(-b)hdef”. Again, one extreme is all the rearranged segments break simultaneously and form the rearranged tumor chromosomes (Figure S4). The other extreme is shown in Figure S4. First, suppose chromosome 5 and 8 are concatenated into “abcdefghij”. The observed tumor chromosome 5 can be created by an inversion of “bcdefg” (also known as reciprocal interchromosomal translocation), an inversion of “(-c)(-b)h”, and finally an inversion of “(-g)(-f)(-e)(-d)(-h)bc” (reciprocal interchromosomal translocation). Note that the term “inversions” generalizes in genome rearrangement theory and encapsulates the genomics term “interchromosomal translocation”. Also, the example demonstrates both scenarios equally explain the same rearranged chromosome.

3.4 What can and cannot be inferred from a complex rearranged genome

We are ready to tie together claims from the previous discussion.

1. *Available statistical evidence does not distinguish a single scenario of copy number neutral events (e.g., chromothripsis) as the source of genome rearrangement.*
2. *Explaining a rearranged genome using only inversions does not mean that inversions are the cause of the rearrangement. Instead, it suggests that an abundance of copy neutral events created the rearrangement.*
3. *Similarly, the number of inversion events is not representative of actual number of rearrangement events that occurred.*
4. *The copy neutral events are best generalized as k-break operations. In this scenario, inversions are 2-breaks, transpositions, and inverted transpositions are 3 breaks, and chromothripsis is the extreme example of an n-break. All combinations can generate complex rearranged genome, and deciding among those options needs additional evidence.*

4 Formal description of algorithms

Stephens et al. simulation Observed breakpoints are supplied as input to the simulation. In the process, breakpoints are added to a single chromosome in random order.

Each breakpoint is two positions with strandedness of the segments adjacent to the positions. B is a set of breakpoints. Assume all segments are in the forward orientation and there is a parsimonious occurence of rearrangements that create the observed breakpoints. Thus, $(+,-),(-,+),(-,-),(+,+)$ breakpoint strandedness correspond with deletion, tandem duplication, tail-to-tail inversion, and head-to-head inversion. Deletions and tandem duplications change the copy number state of the chromosomal segment between breakpoint positions (chromosome length changes). Inversions only inverted the segment and do not change the copy number state (no chromosome length change).

```
procedure StephenSimulateChrom(B, breaks)
    c ← chromosome segmented by positions of B and each segment is in the forward (F) direction.
    while breaks < b < breaks + 5 do
        rearr ← random pop from B
        c ← c with rearr
        b ← unique or non-unique count of segment copy number changes.
    end while
    return c, b
end procedure
```

The simulation is repeated 35 times for copy number state changes of $step(5, 85, 5)$.

Kinsella et al. simulation Chromosomes are simulated by starting with an interval and adding different rearrangements occuring at different rates with number of rearrangements and rearrangement sizes dictated by input distributions.

```
procedure KinsellaSimulateChrom(chromosome-length, rearr-num-distr, rearr-size-distr, inv-rate, del-rate,
dup-rate, tandem-dup-rate, invert-dup-rate)
    ▷ inv-rate, del-rate, dup-rate add up to 1.
    ▷ tandem-dup-rate and invert-dup-rate are 0 − 1
    breaks ← draw from rearr-num-distr
    c ← [(1, chromosome − length), ].
    for i = 1 . . . breaks do
        rearr ← Inversion, Deletion, or Duplication randomly according to rearr rates and choose a random position.
        rearr − size ← draw from rearr-size-distr
        tandem − dup, invert − dup ← if Duplication randomly choose tandem or non-tandem and inverted or not
inverted
        c ← splits 1 or 2 intervals in c based on rearr position and rearr − size.
        c ← delete, duplicate, and invert segments of c according the rearr
    end for
    b ← unique or non-unique count of segment copy number changes.
    return c, b
end procedure
```

Kinsella et al. grimace Show an observed chromosome, supposed to have been shattered, can be created by progressive rearrangements. First, we convert a set of breakpoints into a signed permutation of blocks with respect to a reference genome (i.e. synteny blocks). The signed permutation is then given to GRIMM to resolve a series of progressive rearrangements to reconstruct the reference genome.

For SNU-C1, breakpoints lie within a 80MB region (79749024 bp). Resulting blocks only cover a 40MB region (39652551 bp). No chromosome had two breakpoints with overlapping positions.

procedure PATELBREAKPOINTSTOPERMUTATIONS(*breakpoints*)
  ▷ Partition chromosome by breakpoint positions
  $breakpoints \leftarrow \{(p_i, e_i), (p_j, e_j)$ where $p_i, p_j \in \{1 \ldots L\}$ and $e_i, e_j \in \{\text{head}, \text{tail}\}\}$
  $V \leftarrow (1, \text{head}), (L, \text{tail})$
  $V \leftarrow V \cup (p, \text{tail}), (p, \text{head}) \forall p \in$ breakpoints
  $E \leftarrow breakpoints \cup (p_{i-1}, \text{head}), (p_i, \text{tail}) | 1 \leq i \leq 2n$
  $G(V, E) \leftarrow$ incomplete breakpoint graph. Nodes have 1 or 2 edges.

  $Paths \leftarrow []$
  for $G(B, P) \leftarrow ConnectedComponentsSubgraphs(G(V, E))$ **do** ▷ $B \subset V, P \subset E$
    if $|P| == 1$ **then**
      P is a deleted segment in the rearranged chromosome
      continue
    end if
    if only two nodes $s, t \in B$ have exactly 1 neighbor in G **then**
      ▷ Subgraph is a path starting from s and ending at t
      $Paths \leftarrow DFS(G(V, E), s)$ ▷ list of edges
    else
      $G(B, P)$ is a simple cycle ▷ tandem duplication
    end if
  end for
  ▷ Assemble complete path from $(1, \text{head})$ to (L, tail)
  $StartPath \leftarrow path$ has $(1, \text{head}) \in Paths$
  $EndPath \leftarrow path$ has $(L, \text{tail}) \in Paths$
  $Paths \leftarrow$ randomize ordering of $Paths$
  ▷ Every *path* starts with segment edge, alternates with breakpoint edges, and ends with a segment edge
  $SuperPath \leftarrow Chain(StartPath, Paths - \{StartPath, EndPath\}, EndPath)$

  $L[e] \leftarrow$ label segment e ▷ order segment edges by lowest position and label $1, \ldots, n$
  $L[(p_{i+1}, \text{tail}), (p_i, \text{head})] \leftarrow -L[(p_i, \text{head}), (p_{i+1}, \text{tail})]$
  return $L[e]$ for each segment e in $SuperPath$
end procedure
