# Supplementary figures and images for: The elusive evidence for chromothripsis
Source: Nucleic Acids Res. 2014 Jun 17;42(13):8231–42. doi: 10.1093/nar/gku525 (PMC4117757; doi:10.1093/nar/gku525)

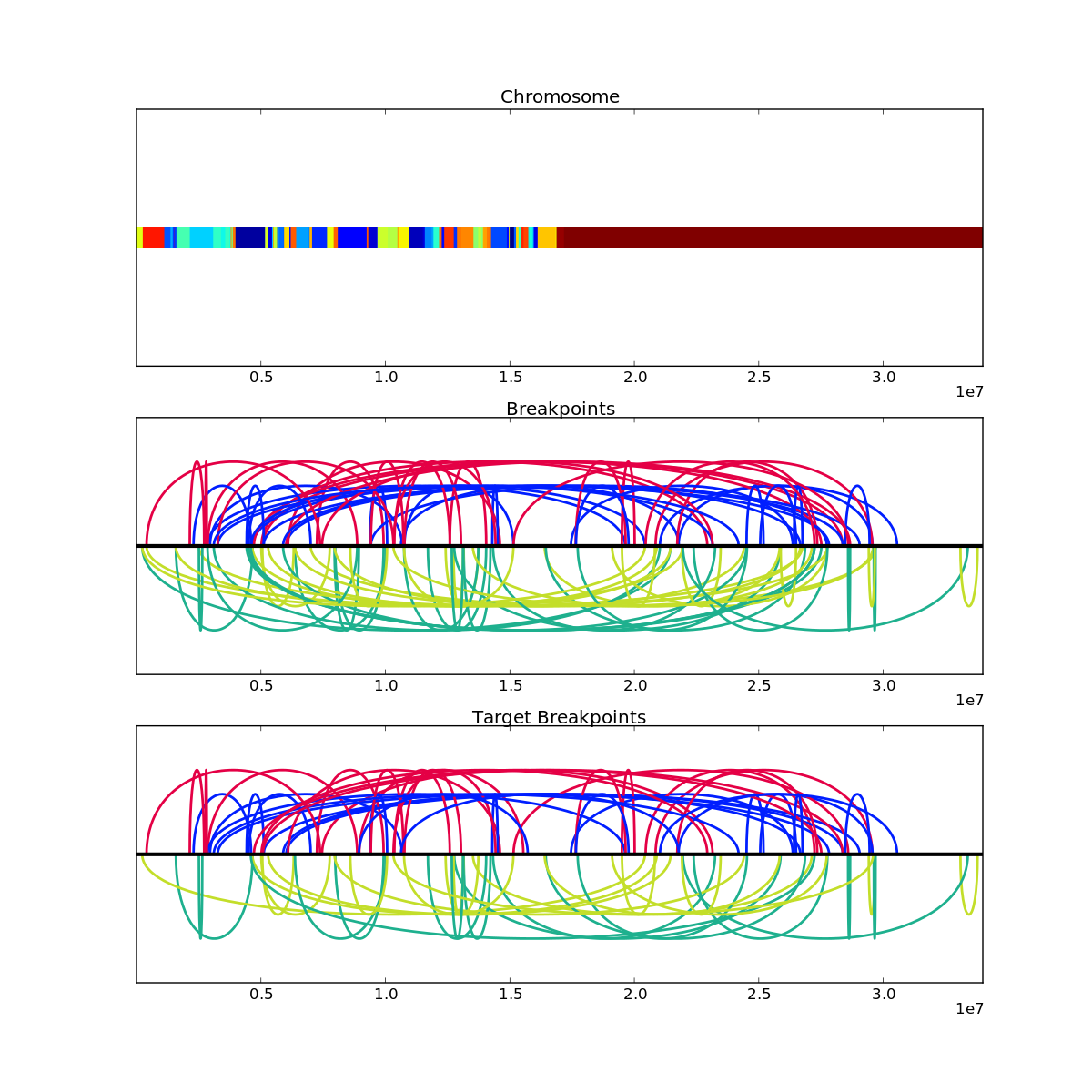

Supplement: SUPPLEMENTARY DATA [file supp_gku525_nar-00163-n-2014-File007.gz › data/8505C_last_frame.png]

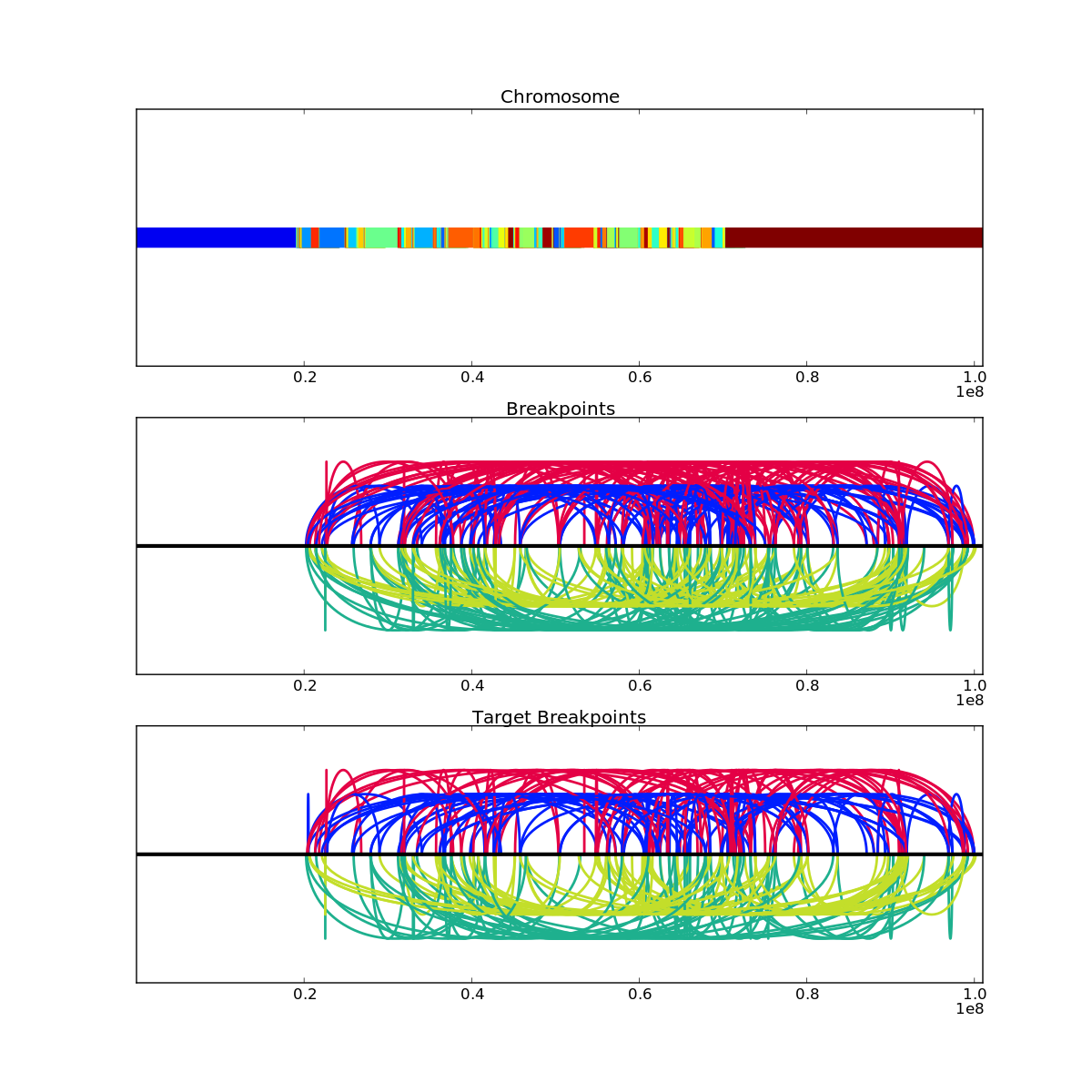

Supplement: SUPPLEMENTARY DATA [file supp_gku525_nar-00163-n-2014-File007.gz › data/SNU-C1_last_frame.png]

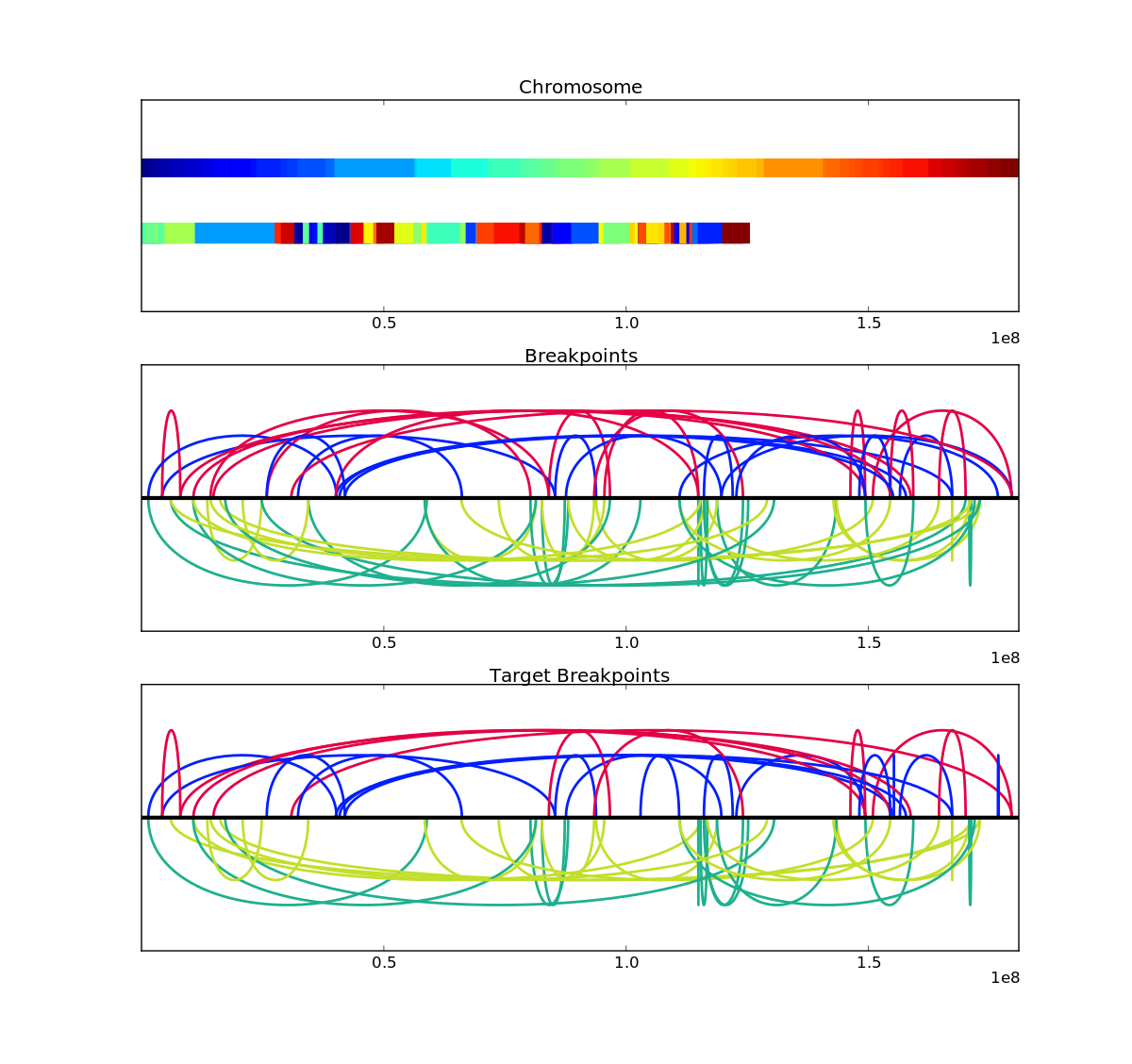

Supplement: SUPPLEMENTARY DATA [file supp_gku525_nar-00163-n-2014-File007.gz › data/TK10_last_frame.png]
